# A Component Prediction Method for Flue Gas of Natural Gas Combustion Based on Nonlinear Partial Least Squares Method

**DOI:** 10.1155/2014/418674

**Published:** 2014-03-19

**Authors:** Hui Cao, Xingyu Yan, Yaojiang Li, Yanxia Wang, Yan Zhou, Sanchun Yang

**Affiliations:** ^1^State Key Laboratory of Electrical Insulation and Power Equipment, School of Electrical Engineering, Xi'an Jiaotong University, Xi'an 710049, China; ^2^School of Energy & Power Engineering, Xi'an Jiaotong University, Xi'an 710049, China

## Abstract

Quantitative analysis for the flue gas of natural gas-fired generator is significant for energy conservation and emission reduction. The traditional partial least squares method may not deal with the nonlinear problems effectively. In the paper, a nonlinear partial least squares method with extended input based on radial basis function neural network (RBFNN) is used for components prediction of flue gas. For the proposed method, the original independent input matrix is the input of RBFNN and the outputs of hidden layer nodes of RBFNN are the extension term of the original independent input matrix. Then, the partial least squares regression is performed on the extended input matrix and the output matrix to establish the components prediction model of flue gas. A near-infrared spectral dataset of flue gas of natural gas combustion is used for estimating the effectiveness of the proposed method compared with PLS. The experiments results show that the root-mean-square errors of prediction values of the proposed method for methane, carbon monoxide, and carbon dioxide are, respectively, reduced by 4.74%, 21.76%, and 5.32% compared to those of PLS. Hence, the proposed method has higher predictive capabilities and better robustness.

## 1. Introduction

Gas-fired generator usually uses the natural gas with high calorific value to generate electricity [1]. Since the natural gas-fired generator is more cost-effective and has less emission compared with coal-fired generator, it is considered as a new type of generator that gives a powerful response to the demand of environmental protection and new market environment. China has abundant domestic natural gas reserves, and the prospect of natural gas generator is bright and broad. Flue gas produced by the natural gas generator mainly consists of methane (CH_4_), carbon monoxide (CO), and carbon dioxide (CO_2_) that will pollute the atmosphere under a certain extent [2]. Therefore, the prediction of components of the flue gas could reflect the degree of the possible impact on the environment and is of great significance for energy saving and emission reduction.

Traditional quantitative analysis of flue gas is always performed by the regular chemical experiment method, which is slow and cumbersome in analysis and may be limited for further application in continuous monitoring of flue gas. Near-infrared spectroscopy (NIR) technology is an effective and rapid quantitative detection method for determining the chemical components based on the characteristic spectrum. NIR has been adopted in medical affairs, food, industries, and other fields [3–6], and it can also be used to realize the component prediction of flue gas. Partial least squares (PLS) is a widely used method in NIR quantitative analysis. PLS synthetically extracts the information for the independent variables and determines the latent variables which have the best interpretability for regression modeling [7]. Since PLS could deal with the multiple correlation among the variables, the accuracy of the regression model would be improved [8–10]. Nevertheless, PLS is essentially a kind of linear regression and may not solve the nonlinear problems effectively [11, 12]. Nonlinear PLS (NPLS) with extended input could solve the problem. For the method, the nonlinear-related independent input matrix is extended to include the nonlinear terms of the independent variables (such as the square terms and the cross-product terms). Then, the PLS regression is performed on the extended input matrix of independent variables [13–15]. Because the spectral data often have thousands of wavelength variables, the extension of nonlinear terms would make the input matrix tremendously large and the nonlinear terms selection also lacks the theoretical basis. Hence, the method is restricted in the application of spectral quantitative analysis. Neural network could approximate a nonlinear function in an arbitrary precision [16–18] and could be used for the extension of the input matrix.

In this paper, a component prediction method which combines the neural network and NPLS with extended input is proposed for flue gas of natural gas combustion. The proposed method uses radial basis function neural network (RBFNN) to extend the input matrix. The original independent input matrix is the input of RBFNN and the outputs of hidden layer nodes of RBFNN are the extension term of the original independent input matrix. Then, the PLS regression is performed on the extended input matrix and the output matrix to establish the NPLS model based on RBFNN extending input (RBFEI-PLS). In order to verify the effectiveness of the proposed method, PLS and RBFEI-PLS are used for building the components quantitative analysis models of the NIR spectral dataset of flue gas of natural gas combustion and the experiments results are analyzed. The organization of the paper is as follows.  Section 2 presents the proposed method. The experimental process is provided and the experiments results are discussed in Section 3. Finally, Section 4 concludes the paper.

## 2. Component Prediction Method

For the NIR spectral dataset of flue gas of natural gas combustion, the absorbance of each wavelength is the independent input **X** and the concentration of the component of flue gas is the dependent output **Y**.

RBFEI-PLS uses typical three-layer construction of RBFNN to extend the input matrix **X**. **X** is the input of RBFNN and the outputs of hidden layer nodes of RBFNN are the extension term **G**, so the extended input matrix will be **X**′ = [**X** **G** 1].

The *i*th node input of the hidden layer, *g*_*i*_, is shown as

(1) $$\begin{array}{c} g_{i}\left(x\right)=\exp\left(-\frac{{||x_{j}-c_{i}||}^{2}}{\sigma_{i}^{2}}\right), \end{array}$$

where *c*_*i*_ is the vector of the *i*th node center and can be obtained according to the cluster center after clustering analysis on the spectral dataset [19]. *σ*_*i*_ is the corresponding width parameter and equals the root mean square distance of the nearest *m* hidden layer nodes from the *i*th node center.

PLS regression is performed on the extended input matrix **X** ^**'**^ and the output matrix **Y** with the flue gas spectral training samples. Then, the quantitative analysis model of the flue gas spectral dataset can be obtained and is shown as

(2) $$\begin{array}{c} Y=XA+GH+b^{T}, \end{array}$$

where **A** is the linear weighting matrix of  **X**, **H** is the nonlinear weighting matrix of the outputs of the hidden layer nodes of RBFNN, and **b** is the bias vector.

## 3. Experiments Results

### 3.1. Experiment Data

**Figure 1:**
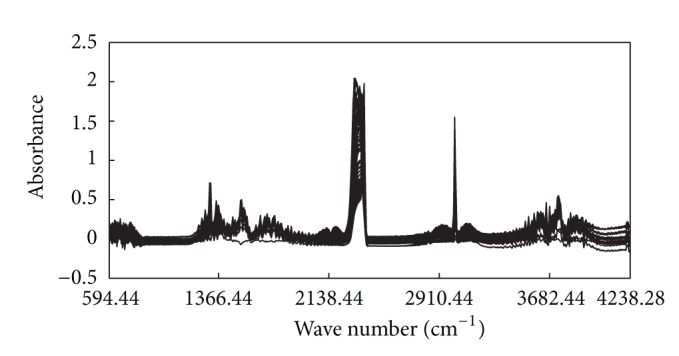
Original spectra data of fuel gas.

To evaluate the effectiveness of the proposed method, a real dataset obtained by measuring the NIR spectra of the field flue gas is used in the experiments. The dataset is obtained during a combustion process and includes 106 samples. Each sample consists of a spectrum for a mixture of CH_4_, CO, and CO_2_. The concentration ranges of the three components, obtained via gas chromatograghy, are 0~0.4598 ppm, 0~0.4083 ppm, and 0~0.3818 ppm, respectively. The spectra were measured by a GASMET DX4000 Fourier transform infrared gas analyzer. The spectral wave number is 549.44~4238.28 cm^−1^ with a resolution of 7.72 cm^−1^. The original spectra are shown in Figure 1.

### 3.2. Experiment Method

The experiment adopts the shutters grouping strategy to divide the original spectral dataset into the calibration set and the validation sets [20] that lets the concentrations of each component vary in roughly the same range. One sample is selected into the validation set every four samples and the rest of the samples are selected into the calibrating set; namely, there are 84 samples in the calibrating set and 21 samples in the validation set. The calibration set is used for building the prediction model of PLS and RBFEI-PLS, and the validation set is used for estimating the effectiveness. For RBFEI-PLS, the number of nodes of the hidden layer is 3, and *m* is set to 3. For PLS and RBFEI-PLS, the number of latent variables is determined according to the root-mean-square error of five-fold cross-validation (RMSECV). In the study, RMSECV, the squared cross-validation correlation coefficient (*R*_cv_^2^), the root mean-squared error of calibration (RMSEC), the squared correlation coefficient of calibration (*R*_*c*_^2^), the root-mean-square error of prediction (RSMEP), and the squared correlation coefficient of prediction (*R*_*p*_^2^) are used to compare the predictive ability of various models.

### 3.3. Results and Analysis

**Figure 2:**
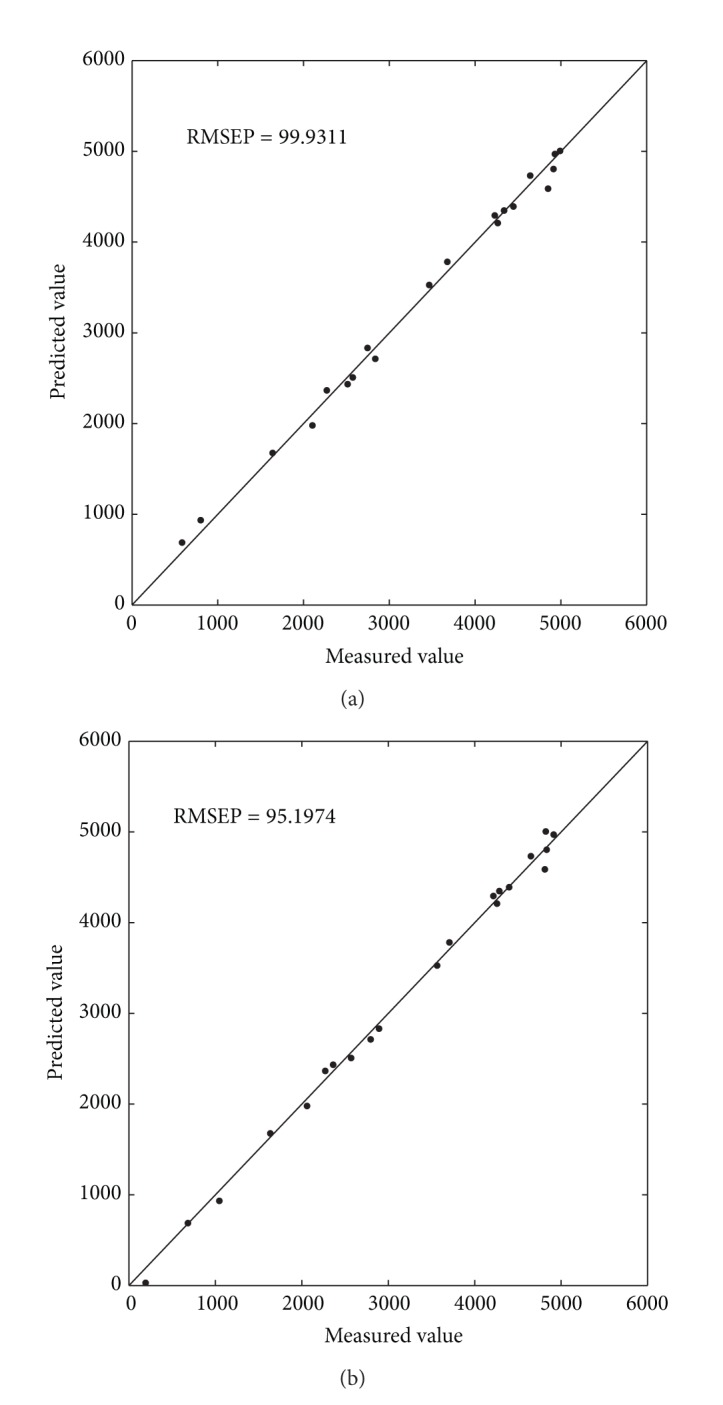
Prediction value versus measured value scatter diagram of different methods for CH_4_. (a) PLS; (b) RBFEI-PLS.

Table 1 is the experiments results for CH_4_ of the flue gas dataset. Although the RMSECV of PLS is smaller, other indexes in Table 1 show that the effectiveness of RBFEI-PLS is much better than that of PLS, especially that the RMSEP value of RBFEI-PLS is 4.74% lower than that of PLS.  Figure 2 shows the scatter plots of measured value versus predicted value of PLS and RBFEI-PLS for CH_4_. PLS has larger error and most points are distributed on both sides of the diagonal line shown in Figure 2(a). Almost all of the points of RBFEI-PLS are in the diagonal line as Figure 2(b) shows. So the prediction capability of RBFEI-PLS is higher.

**Figure 3:**
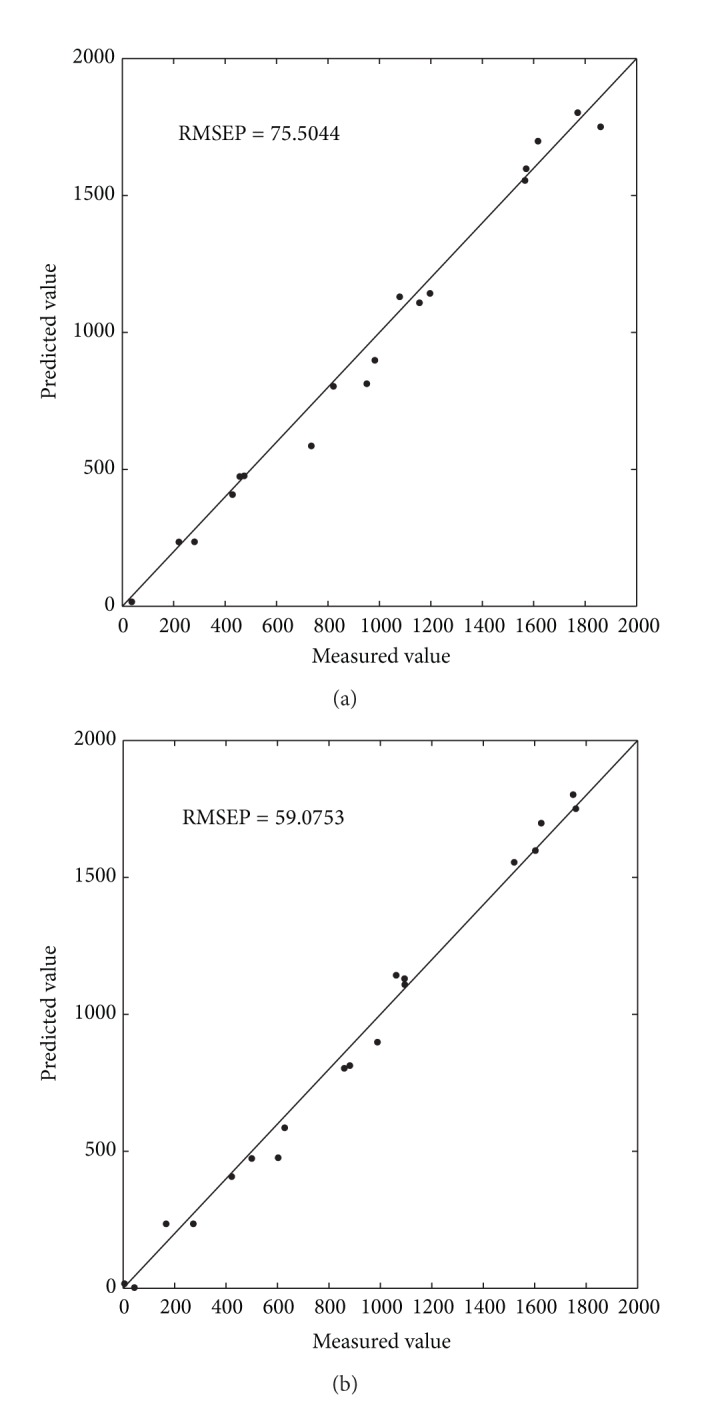
Prediction value versus measured value scatter diagram of different methods for CO. (a) PLS; (b) RBFEI-PLS.

Table 2 is the experiments results for CO of the flue gas dataset. Although the RMSECV value of PLS is smaller and *R*_cv_^2^ of PLS is larger, RBFEI-PLS outperforms PLS according to RMSEC, *R*_*c*_^2^, RSMEP, and *R*_*p*_^2^, where the RMSEP value of RBFEI-PLS is 21.76% lower than that of PLS.  Figure 3 shows the scatter plots of measured value versus predicted value of PLS and RBFEI-PLS for CO. Considerable errors in PLS method are shown in Figure 3(a) as most points are relatively far from the diagonal line, and some points even break away from the diagonal line.  Figure 3(b) indicates that the points are mostly distributed closely on both sides of the diagonal line. Therefore, the prediction model of RBFEI-PLS is still more accurate.

**Figure 4:**
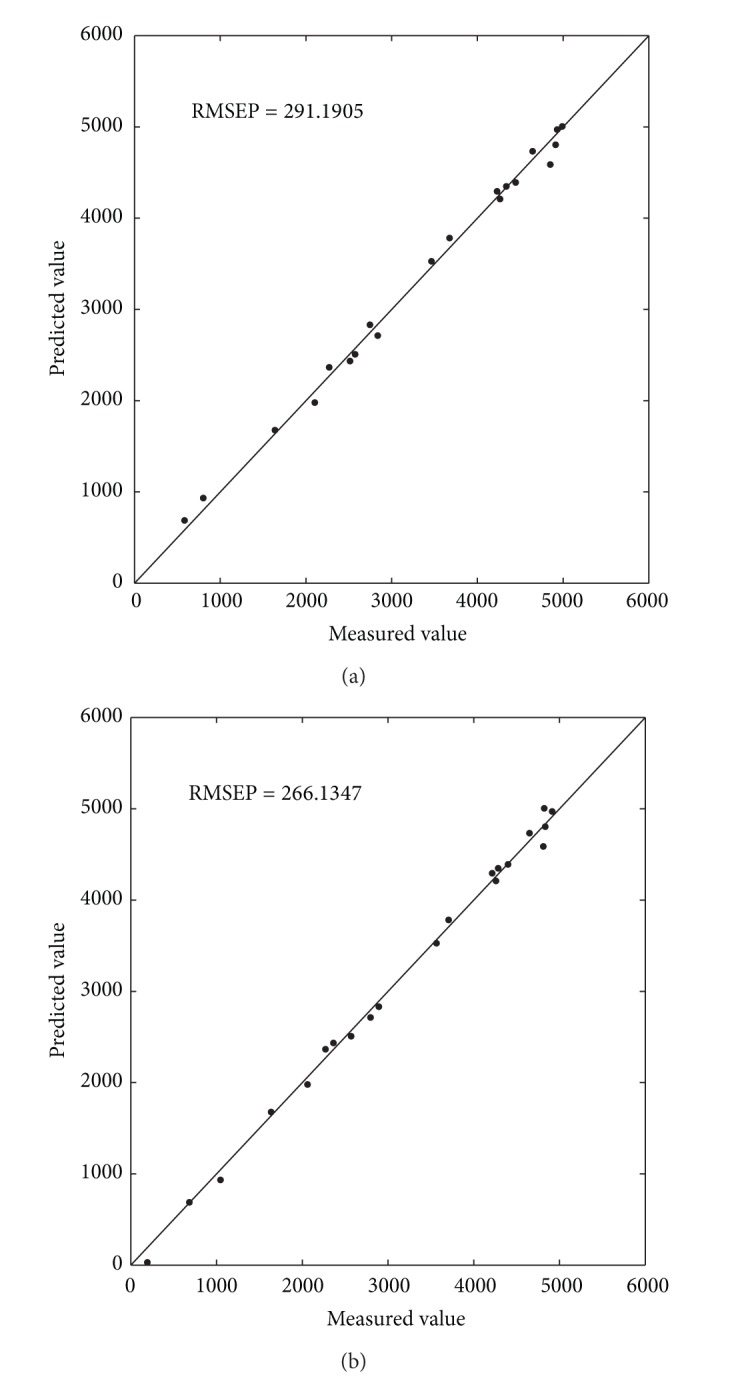
Prediction value versus measured value scatter diagram of different methods for CO_2_. (a) PLS; (b) RBFEI-PLS.

Table 3 is the experiments results for CO_2_ of the flue gas dataset. All the indexes of RBFEI-PLS are better than those of PLS, and the RMSEP value of RBFEI-PLS is 5.32% lower than that of PLS.  Figure 4 shows the scatter plots of measured value versus predicted value of PLS and RBFEI-PLS for CO_2_.  Figure 4(a) shows that most points are distributed on both sides of the diagonal line and some points are even distant from the diagonal line. In Figure 4(b), most points of RBFEI-PLS lie on the diagonal line. As a result, the RBFEI-PLS method has a higher predictive accuracy.

In summary, the experiments results verify that RBFEI-PLS could be adopted for quantitative analysis of flue gas of natural gas combustion successfully and has higher predictive capability.

## 4. Conclusions

In this paper, a component prediction method which combines RBFNN and NPLS with extended input is proposed for component prediction of flue gas of natural gas combustion. Since the proposed method uses RBFNN to extend the input matrix, the nonlinear-related spectral data could be dealt with for quantitative analysis. The experiments results verify that the prediction capability of the proposed method is higher for various components of the flue gas and that the RMSEP values of the proposed method for methane, carbon monoxide, and carbon dioxide are, respectively, reduced by 4.74%, 21.76%, and 5.32% compared to those of PLS. Therefore, the proposed method is an accurate and practical component prediction method for flue gas of natural gas combustion and could be applied for spectral analyses of other analysts.

## Figures and Tables

**Table 1:** Experiment results for CH_4_.

	PLS	RBFEI-PLS
RMSECV	**243.79**	324.28
*R*_cv_^2^	0.7693	**0.9778**
RMSEC	81.1883	**61.8243**
*R*_*c*_^2^	0.997	**0.9983**
RMSEP	99.9311	**95.1974**
*R*_*p*_^2^	0.9960	**0.9963**

**Table 2:** Experiment results for CO.

	PLS	RBFEI-PLS
RMSECV	**241.12**	250.77
*R*_cv_^2^	**0.9358**	0.9354
RMSEC	40.3678	**28.2267**
*R*_*c*_^2^	0.9959	**0.9980**
RMSEP	75.5044	**59.0753**
*R*_*p*_^2^	0.98630	**0.9906**

**Table 3:** Experiment results for CO_2_.

	PLS	RBFEI-PLS
RMSECV	241.12	**154.12**
*R*_cv_^2^	0.5010	**0.9895**
RMSEC	42.5139	**21.7759**
*R*_*c*_^2^	0.9982	**0.9995**
RMSEP	69.5170	**65.8171**
*R*_*p*_^2^	0.9965	**0.9968**
